# PLCG2 is associated with the inflammatory response and is induced by amyloid plaques in Alzheimer’s disease

**DOI:** 10.1186/s13073-022-01022-0

**Published:** 2022-02-18

**Authors:** Andy P. Tsai, Chuanpeng Dong, Peter Bor-Chian Lin, Evan J. Messenger, Brad T. Casali, Miguel Moutinho, Yunlong Liu, Adrian L. Oblak, Bruce T. Lamb, Gary E. Landreth, Stephanie J. Bissel, Kwangsik Nho

**Affiliations:** 1 Stark Neurosciences Research Institute, Indiana University School of Medicine, Indianapolis, IN USA; 2 Department of Medical and Molecular Genetics, Center for Computational Biology and Bioinformatics, Indiana University School of Medicine, Indianapolis, IN USA; 3 Northeast Ohio Medical University, Rootstown, OH USA; 4 Department of Radiology & Imaging Sciences, Indiana University School of Medicine, Indianapolis, IN USA; 5 Department of Medical and Molecular Genetics, Indiana University School of Medicine, Indianapolis, IN USA; 6 Department of Anatomy and Cell Biology, Indiana University School of Medicine, Indianapolis, IN USA

**Keywords:** Alzheimer’s disease, Microglia, PLCG2, Inflammatory response, Co-expression network analysis, Single-cell RNA-Seq analysis

## Abstract

**Background:**

Alzheimer’s disease (AD) is characterized by robust microgliosis and phenotypic changes that accompany disease pathogenesis. Accumulating evidence from genetic studies suggests the importance of phospholipase C γ 2 (PLCG2) in late-onset AD (LOAD) pathophysiology. However, the role of *PLCG2* in AD is still poorly understood.

**Methods:**

Using bulk RNA-Seq (*N*=1249) data from the Accelerating Medicines Partnership-Alzheimer’s Disease Consortium (AMP-AD), we investigated whether *PLCG2* expression increased in the brains of LOAD patients. We also evaluated the relationship between *PLCG2* expression levels, amyloid plaque density, and expression levels of microglia specific markers (*AIF1 and TMEM119*). Finally, we investigated the longitudinal changes of *PLCG2* expression in the 5xFAD mouse model of AD. To further understand the role of *PLCG2* in different signaling pathways, differential gene expression and co-expression network analyses were performed using bulk RNA-Seq and microglial single-cell RNA-Seq data. To substantiate the human analyses, we performed differential gene expression analysis on wild-type (WT) and inactivated *Plcg2* mice and used immunostaining to determine if the differentially expressed genes/pathways were altered by microglial cell coverage or morphology.

**Results:**

We observed significant upregulation of *PLCG2* expression in three brain regions of LOAD patients and significant positive correlation of *PLCG2* expression with amyloid plaque density. These findings in the human brain were validated in the 5xFAD amyloid mouse model, which showed disease progression-dependent increases in *Plcg2* expression associated with amyloid pathology. Of note, increased *Plcg2* expression levels in 5xFAD mice were abolished by reducing microglia. Furthermore, using bulk RNA-Seq data, we performed differential expression analysis by comparing cognitively normal older adults (CN) with 75th percentile (high) and 25th percentile (low) *PLCG2* gene expression levels to identify pathways related to inflammation and the inflammatory response. The findings in the human brain were validated by differential expression analyses between WT and *plcg2* inactivated mice. *PLCG2* co-expression network analysis of microglial single-cell RNA-Seq data identified pathways related to the inflammatory response including regulation of I-kappaB/NF-kappa B signaling and response to lipopolysaccharide.

**Conclusions:**

Our results provide further evidence that *PLCG2* plays an important role in AD pathophysiology and may be a potential target for microglia-targeted AD therapies.

**Supplementary Information:**

The online version contains supplementary material available at 10.1186/s13073-022-01022-0.

## Background

Late-onset Alzheimer’s disease (LOAD) is the most common form of Alzheimer’s disease (AD), with symptoms arising after age 65 [1]. Age, sex, and the apolipoprotein ε4 allele (*APOE* ε4) are the three greatest risk factors for LOAD [2–4]. LOAD risk doubles every 5 years after age 65, and over 60% of AD patients are female [2, 5]. Recent large-scale genome-wide association studies (GWAS) have identified more than 30 genetic loci associated with LOAD, including *APOE*, which is a well-established genetic risk factor [6, 7], but the mechanisms underlying the development of LOAD are incompletely understood.

Importantly, approximately 40% of the susceptibility genes identified in genetic studies of LOAD are immune- and microglia-related, suggesting that microglia are involved in modulating AD pathology [8]. To date, it is known that phospholipase C γ 2 (*PLCG2)* might be important in AD due to the pervious findings that a hypermorphic variant in *PLCG2*, rs72824905, is protective against AD risk [9–11]. However, the role of PLCG2 has not yet been comprehensively explored. PLCG2 is a membrane-associated enzyme that catalyzes the conversion of phospholipid PIP2 (1-phosphatidyl-1D-myo-inositol 4,5-bisphosphate) to IP3 (1D-myo-inositol 1,4,5-trisphosphate) and DAG (diacylglycerol), which plays a crucial role in cell-surface receptor signal transduction [12]. In AD, the effect of *PLCG2* might depend on the stage of neurodegeneration, the gene expression, the enzymatic activity, and the formation of intracellular signaling complexes in microglia [10, 13–16]. Therefore, a better understanding of the role of *PLCG2* in AD and microglia is needed for the development of *PLCG2*-directed therapeutics.

Here, we performed differential gene expression and gene-set enrichment analysis of transcriptomic data, including single-cell RNA-Seq data, from human and mouse brain regions to investigate the role of *PLCG2* in AD. Expression levels of *PLCG2* were increased in LOAD compared to those in cognitively normal older adults (CN) and were positively associated with both amyloid plaques and expression levels of microglial marker genes. Similarly, in a 5xFAD mouse model, *Plcg2* expression increased in a disease progression-dependent manner and *Plcg2* was highly expressed in plaque-induced microglia in the brain. Importantly, differential expression analysis in CN and a *Plcg2*-inactivated mouse model identified inflammation-related pathways, suggesting a potential role for *PLCG2* in inflammation-related pathways. Notably, the differentially expressed genes/pathways were not altered by microglial cell coverage or morphology, *PLCG2* co-expression network analysis of microglial single-cell RNA-Seq data identified pathways related to the inflammatory response, including regulation of I-kappaB/NF-kappa B signaling, and response to lipopolysaccharide.

Overall, our results suggest that *PLCG2* plays an important role in the inflammation-related pathway in AD and that *PLCG2* might be a microglia-associated potential therapeutic target in AD treatment.

## Methods

### Bulk RNA-Seq data analysis in the post-mortem human brain

RNA-Seq data were downloaded from the Accelerating Medicines Partnership for Alzheimer’s Disease (AMP-AD) Consortium (https://adknowledgeportal.synapse.org/Explore/Programs/DetailsPage?Program=AMP-AD) through the Synapse database (https://www.synapse.org/): the Mayo Clinic Brain Bank cohort (Mayo; https://www.synapse.org/#!Synapse:syn8466812), the Mount Sinai Medical Center Brain Bank (MSBB; https://www.synapse.org/#!Synapse:syn8484987) cohort, and the Religious Orders Study and Memory and Aging Project (ROS/MAP; https://www.synapse.org/#!Synapse:syn8456629) cohort (Table 1) [17–19].

**Table 1:**
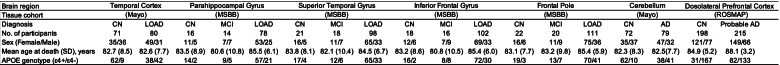
shows the demographic information of the participants included in this study

*TCX* temporal cortex, *PHG parahippocampal gyrus*, *STG superior temporal gyrus*, *IFG inferior temporal gyrus*, *FP frontal pole*, *CER cerebellum*, *DLPFC dorsolateral prefrontal cortex, CN cognitively normal, AD Alzheimer’s disease, MCI mild cognitive impairment, RIN RNA integrity number, APOE ε4+/- carriers and non-carriers of the APOE ε4 allele*

In the Mayo Clinic RNA-Seq dataset, the RNA-seq-based whole transcriptome data were generated from the temporal cortex and cerebellum. In the MSBB dataset, data were generated from parahippocampal gyrus, inferior frontal gyrus, superior temporal gyrus, and frontal pole. In the ROSMAP dataset, the RNA-seq data were generated from dorsolateral prefrontal cortices. The procedures for sample collection, post-mortem sample descriptions, tissue and RNA preparation methods, library preparation and sequencing methods, and sample quality controls were previously described in detail [18–22].

To investigate the diagnostic group difference in *PLCG2* expression among CN, MCI, and those with LOAD, we used the *limma* software [23] to perform differential expression analysis [21]. Age, sex, and *APOE* ε4 carrier status were used as covariates. To investigate the relationship between *PLCG2* expression levels and amyloid plaque density (number of plaques/mm^2^) [19, 22] and expression levels of microglia-specific markers (*AIF1 and TMEM119)*, we used linear regression models with *PLCG2* expression levels as a dependent variable and plaque density or microglial-specific markers and age, sex, and *APOE* ε4 carrier status as explanatory variables. The regression was performed with the “glm” function of the stats package in R (version 3.6.1).

The expression levels of *PLCG2*, *AIF1*, and *TMEM119* were downloaded from the Synapse database, and the log2 counts-per-million (logCPM) normalized data of RNA-seq were used for the following analysis in this study.

### Microglia single-cell RNA-Seq data analysis

Single-cell transcriptomic data from human microglia isolated from fresh autopsy tissue were download from the AMP-AD consortium database, for which the raw data are accessible through Synapse (https://www.synapse.org/#!Synapse:syn21438358) [24]. Brain myeloid cells isolated from the dorsolateral prefrontal cortex of 12 donors (four mild cognitive impairment (MCI) and eight AD patients) of both sexes from the ROS/MAP cohort were used. Data were processed with cell ranger count (CellRanger version 5.0.1) [24] using a custom reference package based on the human reference genome GRCh38 (Gencode.V32). Subsequent data analysis was carried out using the Seurat package (v 4.0.0) [25]. We further filtered out cells by genes detected per cell (nFeature_RNA) based on whether over 5000 or less than 200 were detected. Dead cells were excluded by retaining cells with less than 5% mitochondrial reads (percent. mt<5%). A total of 15,021 cells were qualified for the following analysis.

Microglia single-cell RNA-Seq analyses were performed by Seurat package in R [25]. Data were normalized using the NormalizeData() function, and the variable features were identified using the FindVariableFeatures() with 2000 genes; the selection method was set to “vst,” a variance-stabilizing transformation. To identify the integration of anchor genes among the 12 samples, the FindIntegrationAnchors() function was used with 20 principal components and 2000 genes. Using Seurat’s IntegrateData() function, samples were combined into one object. The data were scaled using the ScaleData() function to reduce the dimensionality of this dataset; principal component analysis was used, and the first 30 principal components were summarized further using UMAP dimensionality reduction. Clustering was conducted using the FindNeighbors() and FindClusters() functions using 30 principal components and a resolution parameter set to 0.6. Due to the sparse nature of single-cell transcriptomic profiles, an ensemble tree-based algorithm, GENIE3, was used to reconstruct the co-expression module networks of *PLCG2* [26], which calculates the co-expression pattern of PLCG2 and the expression pattern of the other 2469 genes that are expressed in more than 10% of the total number of cells.

### Differential gene expression and pathway enrichment analysis

The limma package in R software was used to identify differentially expressed genes. The ClusterProfiler package was used to automate biological-term classification and enrichment analysis for the upregulated and downregulated genes [27].

### Mice

Wild-type (WT) and 5xFAD mice used for immunofluorescence and qPCR studies were maintained on the C57BL/6J background and purchased from the Jackson Laboratory (JAX MMRRC Stock# 034848). One-, 4-, 6-, and 8-month-old mice were used. In the PLX5622 animal treatment study described in the “Methods” section below, we used WT and 5xFAD mice that were maintained as mixed C57BL/6J and SJL background 5xFAD mice [B6SJL-Tg (APPSwFlLon, PSEN1*M146L*L286V) 6799Vas, Stock #34840-JAX]). The 5xFAD transgenic mice overexpress the following five FAD mutations: the APP (695) transgene containing the Swedish (K670N, M671L), Florida (I716V), and London (V7171) mutations, and the PSEN1 transgene containing the M146L and L286V FAD mutations.

To deplete microglia, both WT and 5xFAD mice were treated PLX5622, a colony-stimulating factor receptor-1 antagonist, at 4 months of age. Either normal rodent diet or PLX5622-containing chow was administered for 28 days. An additional cohort of 4-month-old mice was treated with PLX5622 or the control diet for 28 days, then those initially administered the PLX5622 feed were given a normal rodent diet for an additional 28 days [28]. At 6 months of age, this cohort of mice was euthanized. PLX5622 was provided by Plexxikon formulated as the AIN-7 diet at 1200 mg/kg.

Plcg2 inactivation (PLCG2 ^inact^) mice for differential expression analysis were generated by IU/JAX/UCI MODEL-AD consortium and maintained on the C57BL/6J background (JAX MMRRC Stock# 029910; https://www.model-ad.org/strain-table/). PLCG2 ^inact^ mice were generated by using CRISPR/cas9 endonuclease mediated genome editing to introduce a 13 bp knock-out mutation (nucleotides 1570 to 1582).

Up to five mice were housed per cage with SaniChip bedding and LabDiet® 5K52/5K67 (6% fat) feed. The colony room was kept on a 12:12-h light/dark schedule with the lights on from 7:00 am to 7:00 pm daily. The mice were bred and housed in specific-pathogen-free conditions. Both male and female mice were used, and the numbers of male and female mice were equally distributed. The number of mice used for each experiment is stated in the corresponding figure legends, and the results of individual values are shown in the scatter plot.

Mice were euthanized by perfusion with ice-cold phosphate-buffered saline (PBS) following full anesthetization with Tribromoethanol [Avertin®] (125-250 mg/kg intraperitoneal injection). Animals used in the study were housed in the Stark Neurosciences Research Institute Laboratory Animal Resource Center, Indiana University School of Medicine. All animals were maintained, and experiments performed in accordance with the recommendations in the Guide for the Care and Use of Laboratory Animals of the National Institutes of Health. The protocol was approved by the Institutional Animal Care and Use Committee (IACUC) at Indiana University School of Medicine.

### Mouse RNA isolation for qPCR and NanoString nCounter analysis

The cortical and hippocampal regions were micro-dissected and stored at −80°C. The frozen brain tissue was homogenized in buffer consisting of 20 mM Tris-HCl (pH=7.4), 250 mM sucrose, 0.5 mM ethylene glycol-bis (β-aminoethyl ether)-N,N,N’,N’-tetraacetic acid (EGTA), 0.5 mM ethylenediaminetetraacetic acid (EDTA), and RNase-free water and were stored in an equal volume of RNA-Bee (Amsbio, CS-104B) at −80°C until RNA extraction was performed. RNA was isolated by chloroform extraction and purified using the Purelink RNA Mini Kit (Life Technologies). The cDNA was prepared from 750 ng of RNA using the High-Capacity of RNA-to-cDNA kit (Applied Biosystems), and qPCR was performed on the StepOne Plus Real-Time PCR system (Life Technologies) with the Taqman Gene Expression Assay (Mm01242530_m1, Life Technologies). Relative gene expression was determined with the △△CT method and was assessed relative to GAPDH (Mm99999915_g1). Student’s *t* test was performed for qPCR results comparing WT and 5xFAD animals.

For NanoString nCounter analysis, the neuroinflammation panel was used (catalog numbers XT-CSO-MNROI1-12, Seattle, WA, USA). Two hundred nanograms of RNA was loaded per 1-month-old male mouse from the WT and PLCG2-inactivation mouse samples (*n*=3) and was hybridized with probes for 16 h at 65°C. The results obtained from the nCounter MAX Analysis System (NanoString Technologies, catalog #NCT-SYST-LS, Seattle WA) were imported into the nSolver Analysis Software (v4.0; NanoString Technologies) for QC verification, normalization, and data statistical analysis using Advanced Analysis software (v2.0.115; NanoString Technologies). All assays were performed according to the manufacturer’s protocols [29].

### Immunofluorescence

The brains from mice at 8 months of age were fixed in 4% PFA overnight at 4°C. Following overnight fixation, the brains were cryoprotected in 30% sucrose at 4°C and were embedded. The brains were processed on a microtome as 30-μm free-floating sections. For immunostaining, at least three matched brain sections were used. Free-floating sections were washed and permeabilized in 0.1% TritonX in PBS (PBST), followed by antigen retrieval using 1x Reveal Decloaker (Biocare Medical) at 85°C for 10 min. Sections were blocked in 5% normal donkey serum in PBST for 1 h at room temperature (RT). The sections were incubated in the following primary antibodies in 5% normal donkey serum in PBST overnight at 4°C: IBA1 (Novus Biologicals #NB100-1028, 1:1000); P2RY12 (AnaSpec, #AS-55043A, 1:1000). Sections were washed and visualized using the respective species-specific AlexaFluor fluorescent antibodies (diluted 1:1000 in 5% normal donkey serum in PBST for 1 h at RT). Sections were washed 3 times in PBST for 10 min, then incubated in DAPI for 5 min. Sections were counterstained and mounted onto slides. Images were acquired on a fluorescence microscope with similar exposure and gains across stains and animals. Microglia-morphology was measured as previously described [30] using a Nikon A1R confocal microscope (Nikon Instruments, Melville, NY). Z-stacks (1μm steps) were acquired through layer V of the visual cortex in 5 slices per animal. Images were blinded, and the area coverage of microglia was calculated using ImageJ software [31].

### Statistical analysis

In the mouse study, statistical analyses were performed using GraphPad Prism (Version 8.4.2). Experiments at the 2-, 4-, 6- and 8-month time points were performed independently, and statistical comparisons between WT and 5xFAD AD mice were performed by an unpaired *t* test.

## Results

### PLCG2 expression levels are increased in LOAD, compared to CN

**Fig. 1:**
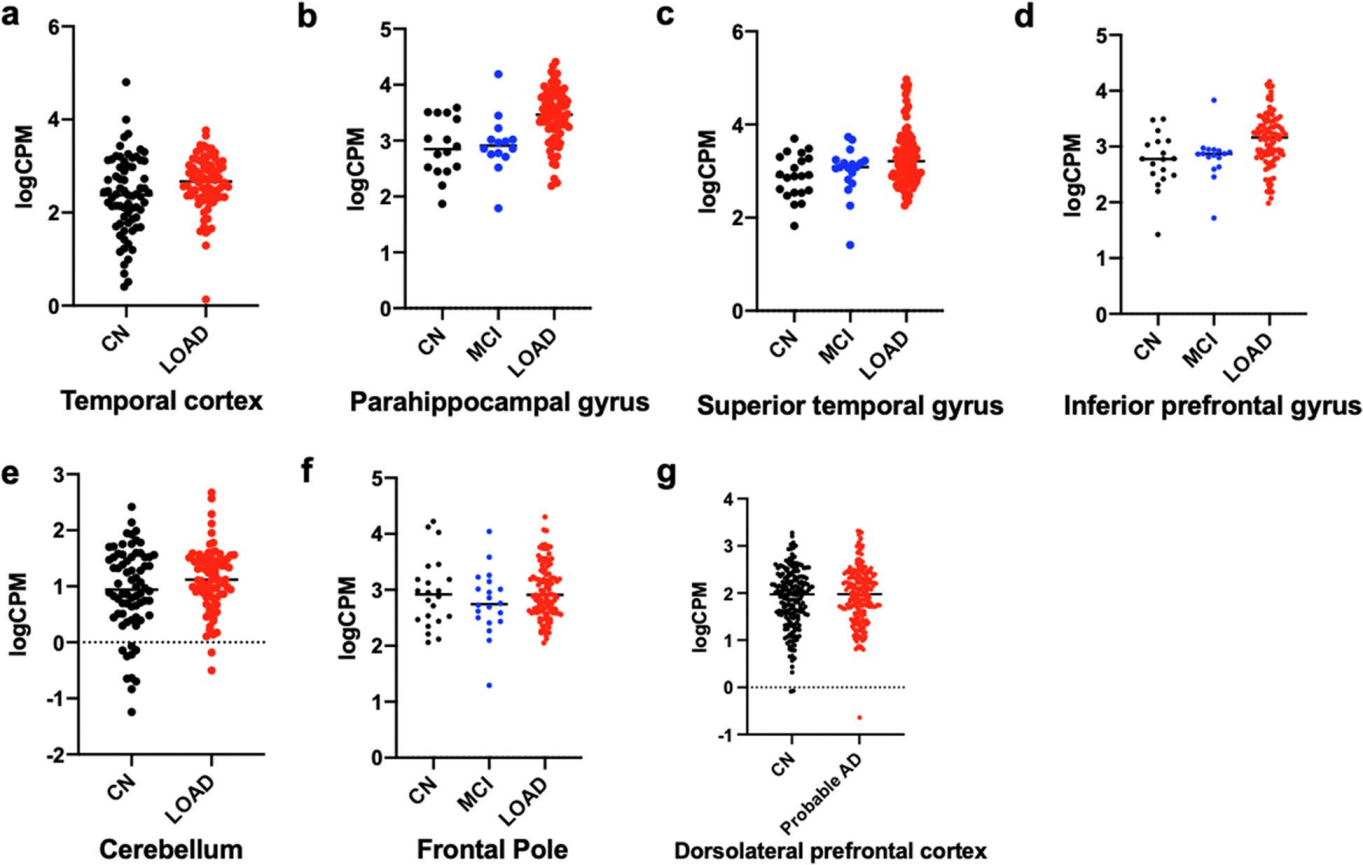
Difference of *PLCG2* expression levels across the diagnostic groups. Gene expression levels are shown as logCPM values. **a** Temporal cortex - Mayo, **b** Parahippocampal gyrus - MSBB, **c** Superior temporal gyrus - MSBB, **d** Inferior prefrontal gyrus - MSBB, **e** Cerebellum - Mayo, **f** Frontal pole - MSBB, and **g** Dorsolateral prefrontal cortex - ROS/MAP. CN cognitively normal older adults, AD Alzheimer’s disease, MCI mild cognitive impairment

The demographic information of participants included in this study is summarized in Table 1. From gene expression analysis with age and sex as covariates, using RNA-Seq data generated from seven brain regions (Additional file 1: Table S1), we found that *PLCG2* was overexpressed in LOAD patients in the temporal cortex (logFC=0.27, *p*=4.56E-02; Fig. 1a), parahippocampal gyrus (logFC=0.55, *p*=1.74E-03; Fig. 1b), superior temporal gyrus (logFC=0.46, p=2.55E-02; Fig. 1c), and inferior prefrontal gyrus (logFC=0.36, *p*=1.38E-02; Fig 1d) (Table 2). However, we did not find any diagnostic group differences in the cerebellum (Fig. 1e), frontal pole (Fig. 1f), and dorsolateral prefrontal cortex (Fig. 1g) (Table 2). With inclusion of *APOE* ε4 carrier status as an additional covariate, *PLCG2* remained overexpressed in LOAD patients in the parahippocampal gyrus (logFC=0.57, *p*=2.02E-03), superior temporal gyrus (logFC=0.42, *p*=4.95E-02), and inferior prefrontal cortex (logFC=0.33, *p*=2.60E-02) (Table 2). In the parahippocampal gyrus, superior temporal gyrus, and inferior prefrontal gyrus, *PLCG2* was overexpressed in LOAD patients with and without *APOE* ε4 carrier status as an additional covariate. We then investigated whether expression levels of *PLCG2* were associated with expression levels of microglia-specific marker genes (*AIF1* and *TMEM119*). Our analysis revealed that *AIF1* and *TMEM119* expression levels were significantly associated with *PLCG2* expression in the frontal pole (*AIF1*: *β*=0.258, p=1.09E-06; *TMEM119*: *β*=0.518, *p*=1.28E-15), superior temporal gyrus (*AIF1*: *β*=0.33, *p*=8.98E-07; *TMEM119*: *β*=0.71, *p*<2E-16), parahippocampal gyrus (*AIF1*: *β*=0.419, *p*=5.43E-07; *TMEM119*: *β*=0.806, *p*<2E-16), and inferior prefrontal gyrus (*AIF1*: *β*=0.257, *p*=6.15E-06; *TMEM119*: *β*=0.582, *p*=6.64E-15) (Table 3).

**Table 2:**
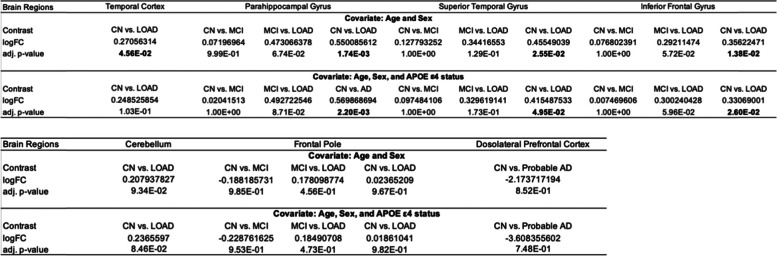
shows the *p*-values from gene expression analysis using RNA-Seq data from the AMP-AD consortium

*TCX* temporal cortex, *PHG parahippocampal gyrus*, *STG superior temporal gyrus*, *IFG inferior temporal gyrus*, *FP frontal pole*, *CER cerebellum*, *DLPFC dorsolateral prefrontal cortex, CN cognitively normal, AD Alzheimer’s disease*, *MCI mild cognitive impairment, logFC log fold-change*

**Table 3:**
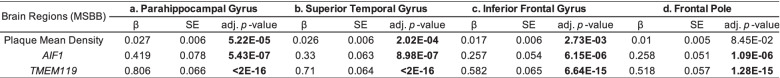
PLCG2 expression levels were associated with amyloid plaque density and expression levels of microglia specific markers Table 3 shows the *β* coefficient (*β*), standard error (SE), and *p*-value for the association analysis between *PLCG2* expression levels and amyloid plaque density or expression levels of microglia specific markers, *AIF1* and *TMEM119* by general linear models

### Increased PLCG2 expression levels are associated with amyloid plaques in the human brain

**Fig. 2:**
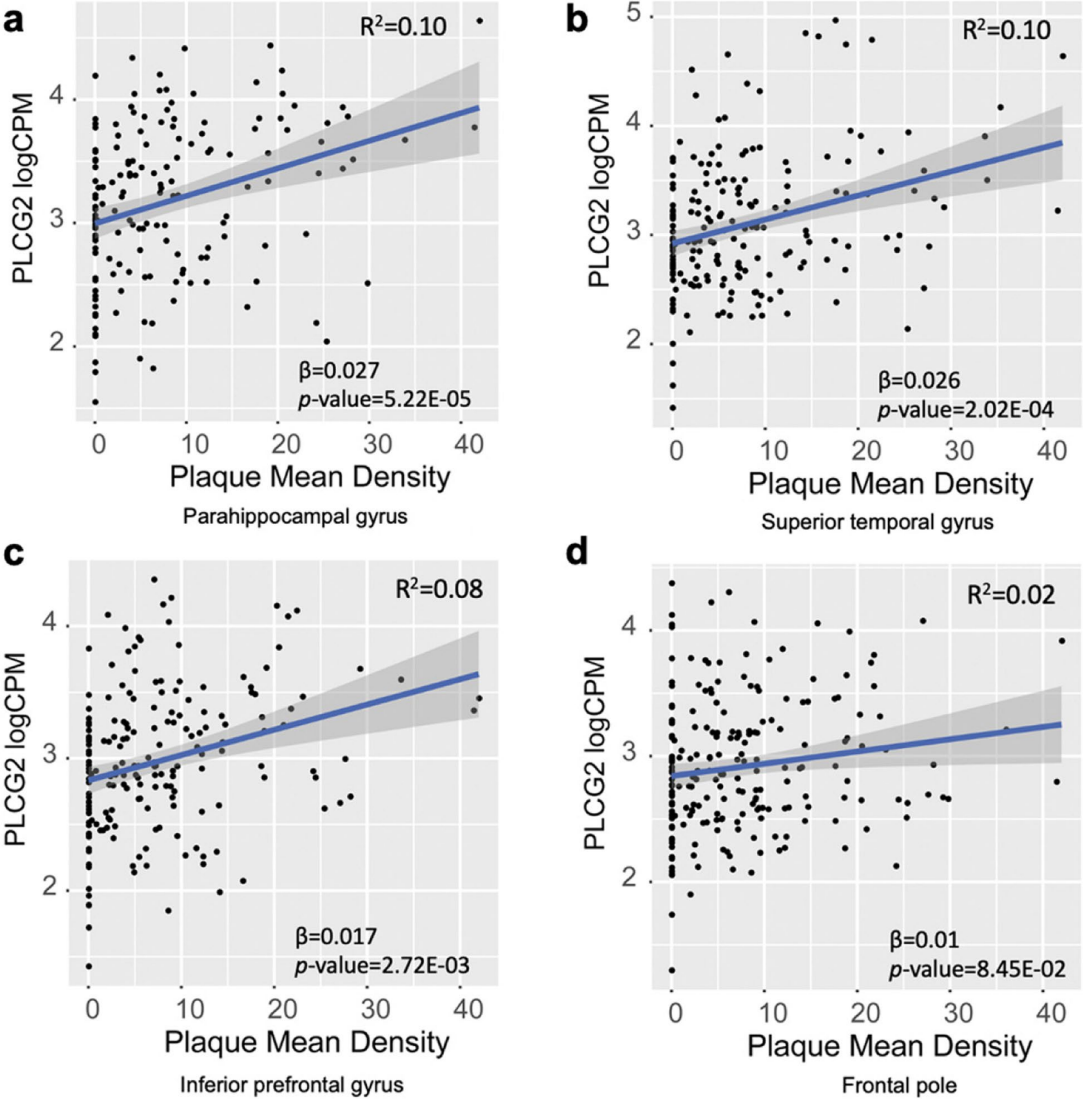
Associations of *PLCG2* expression levels with mean amyloid plaque density. The scatter plots show positive associations between *PLCG2* expression levels and mean plaque density in the **a** parahippocampal gyrus, **b** superior temporal gyrus, and **c** inferior prefrontal gyrus and **d** frontal pole from the MSBB cohort

We performed an association analysis of expression levels of *PLCG2* and mean amyloid plaque densities measured in four brain regions. The *p*-values and beta values were obtained by linear regression in each separate brain region. Expression levels of *PLCG2* in the human brain were associated with amyloid plaques in three brain regions (Table 3 and Additional file 1: Table S2). Increased *PLCG2* expression levels were associated with increased amyloid plaque levels in the parahippocampal gyrus (*β*=0.027, *p*=5.22E-05; Fig. 2a), superior temporal gyrus (*β*=0.026, *p*=2.02E-04; Fig. 2b), and inferior prefrontal cortex (*β*=0.017, *p*=2.73E-03; Fig. 2c), but not in the frontal pole (*β*=0.01, *p*=0.08; Fig. 2d).

### Plcg2 expression is increased exclusively in microglia in the brains of 5xFAD mice

**Fig 3.**
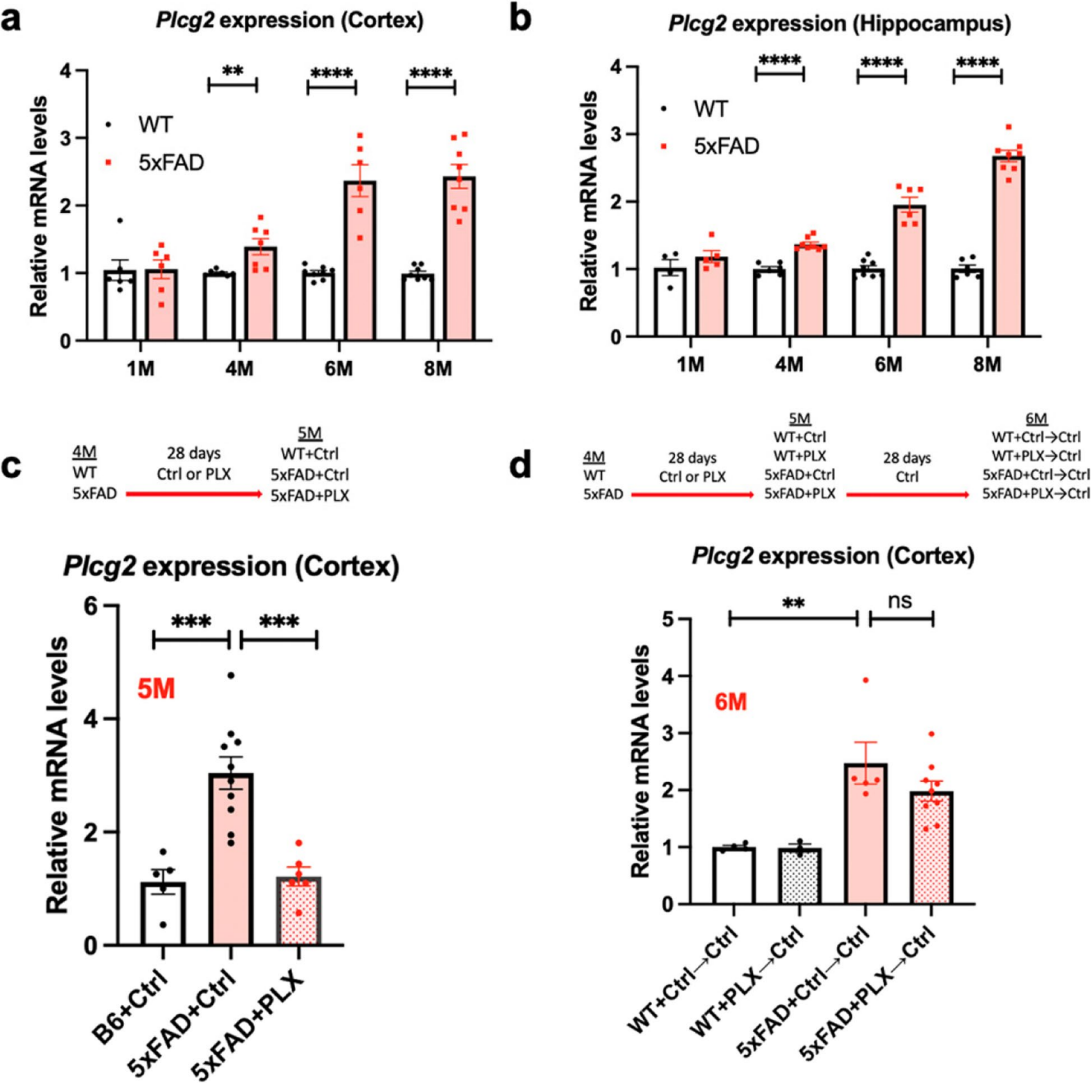
*Plcg2* expression is increased exclusively in microglia in the brains of 5xFAD mice*. Plcg2* levels were assessed in cortical and hippocampal lysates from 5xFAD mice. There were significant changes in *Plcg2* gene expression in both the **a** cortex and **b** hippocampus at 4, 6, and 8 months of age (*n*=4–8 mice). However, increased *Plcg2* expression levels were abolished after the PLX5622 treatment (**c**) and restored after switching the PLX diet to a normal diet (**d**) (*n*=3–10). **p<0.01; ***p<0.001; ****p<0.0001, ns not significant

*Plcg2* expression was increased in the 5xFAD mouse model of amyloid pathology, consistent with our findings in human LOAD. In 5xFAD mice, *Plcg2* expression was increased in the cortex (Fig. 3a) and hippocampus (Fig. 3b) of 4-, 6-, and 8-month-old mice (4-month: 1.39-fold in the cortex and 1.37-fold in the hippocampus; 6-month: 2.37-fold in the cortex and 1.96-fold in the hippocampus; and 8-month: 2.43-fold in the cortex and 2.67-fold in the hippocampus) (Fig. 3). Furthermore, our analysis showed a disease progression-dependent increase in *Plcg2* expression in mice with amyloid pathology. To assess our findings that *PLCG2* expression levels were associated with expression levels of microglia-specific marker genes in the human brain (Table 3 and Additional file 1: Table S2), we depleted microglia in 4-month-old 5xFAD mice by treating the animals with the PLX5622 (PLX), a colony-stimulating factor receptor-1 antagonist, for 28 days [22, 28]. In the cortex, the increase in *Plcg2* expression in 5xFAD mice was reduced to control levels by the PLX treatment, despite only 70% microglial depletion (Fig. 3c). Furthermore, expression levels of *Plcg2* were restored after switching the mouse diet from the PLX diet to a normal diet for 28 further days (Fig. 3d). However, we did not observe any increase in *Plcg2* expression in a 12-month tauopathy mouse model (P301S), which exhibits robust microgliosis (Additional file 2: Fig. S1).

### Differential expression analysis in cognitively normal older adults and Plcg2-inactivated mouse model identified inflammation-related pathways

**Fig. 4:**
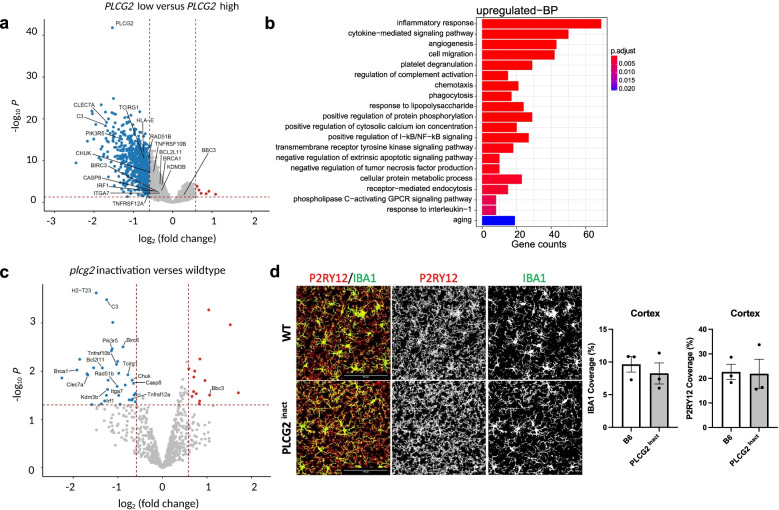
Differential expression analysis in cognitively normal older adults and *Plcg2*- inactivated mouse model identified inflammation-related pathways. The volcano plot shows significant DEGs (fold change>1.5, FDR-corrected *p*<0.05) in cognitively normal older adults (CN) with lower (bottom 25%; *n*=50; low) versus higher (top 25%; *n*=50; high) expression of *PLCG2* (**a**). Gene-set enrichment analysis was performed on the DEGs (fold change>1.5, FDR-corrected *p*<0.05) to identify GO terms, including inflammatory response (**b**). The volcano plot shows significant DEGs derived from the NanoString analysis of the brains of *Plcg2* inactivation (PLCG2 ^inact^, *n*=3) and WT (*n*=3) mice (1-month male) (**c**). Immunostaining of microglial markers with IBA1 (green) and P2RY12 (red) and quantification of percent area in brain sections of 1-month-old male WT and PLCG2 ^inact^ demonstrate unchanged microglial morphology and area coverage (**d**). GO *gene ontology*, BP *biological process,* DEGs *differentially expressed genes*, FDR *false discovery rate*

We performed differential expression analysis of bulk RNA-Seq data from the ROS/MAP cohort by comparing CN in the 25th percentile of *PLCG2* gene expression levels with CN participants in the 75th percentile of *PLCG2* gene expression levels (Additional file 1: Table S3). The analysis identified 5047 significantly differentially expressed genes (FDR-corrected *p*<0.05), including 4566 downregulated (992 genes with larger than 1.5-fold changes) and 481 upregulated genes (6 genes with larger than 1.5-fold changes) (Fig. 4a). Pathway analysis identified several GO terms, including the inflammatory response (FDR-corrected *p*=5.77E-32) (Fig. 4b). To validate our findings in humans, we generated mice with inactivated *Plcg2* and performed differential expression analysis by comparing *Plcg2*-inactivated mice with WT mice at one month of age using gene expression data from the NanoString Neuroinflammation panel (757 genes) (Additional file 1: Table S4). Our analysis identified 74 significantly differentially expressed genes, including 39 downregulated genes (35 genes with larger than 1.5-fold changes) and 35 upregulated genes (14 genes with larger than 1.5-fold changes). Of these genes, 17 genes shown in the volcano plot (16 downregulated and 1 upregulated) were identified to have the same differentially expressed directions in both humans and mice (Fig. 4c). Differentially expressed genes (FDR-corrected *p*<0.05), including *ccl2*, *cx3cl1*, and *cxcl9*, are shown in the heatmap with enrichment analysis of KEGG pathways (Additional file 2: Fig. S2a) and Gene Ontology terms for biological processes (Additional file 2: Fig. S2b), suggesting the role of *PLCG2* in the inflammatory response. To determine if the differentially expressed genes/pathways were altered by microglial cell coverage or morphology, we performed immunostaining. Immunostaining of 1-month-old WT and PLCG2 ^inact^ mice revealed that the microglial cell coverage and morphology were unchanged (Fig. 4d).

### PLCG2 co-expression network analysis of microglial single-cell RNA-Seq data identified inflammatory response-related pathways

**Fig. 5:**
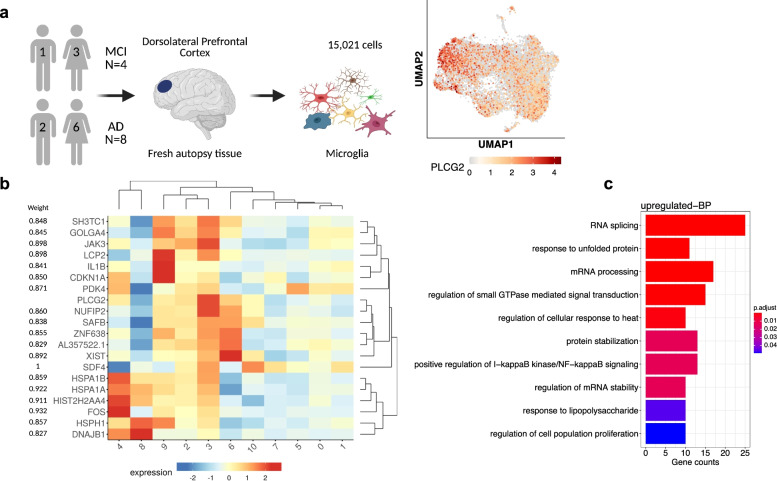
*PLCG2* co-expression network analysis of microglial single-cell RNA-Seq data identified inflammatory response-related pathways. In the microglia single-cell RNA-Seq data, cells in the dorsolateral prefrontal cortex were isolated from 12 donors of both sexes. After quality control procedures, 15,021 cells were used for analysis. The UMAP plot shows the expression of *PLCG2*, and each dot represents a cell (**a**). The heatmap shows gene expression levels of the top 20 genes identified as co-expressed with *PLCG2* using GENIE3 (**b**). The top ten GO terms from gene-set enrichment analysis of the top 20% of upregulated genes co-expressed with *PLCG2* in human microglia in the disease condition (**c**). UMAP *Uniform Manifold Approximation and Projection,* MCI *mild cognitive impairment,* AD *Alzheimer's disease*

We analyzed single-cell RNA-Seq data from 15,021 freshly isolated microglial cells [24]. The visualization of the cells in a UMAP plot showed the separation of the cell expressed *PLCG2* (Fig. 5a). *PLCG2* co-expression network analysis of gene expression profiles using GENIE3 identified the co-occurrence of genes and pathways associated with *PLCG2* in the microglia of MCI and AD patients (Additional file 1: Table S5). Figure 5b shows the top 20 genes that were co-expressed with *PLCG2*. Gene-set enrichment analysis of the top 20% of upregulated genes co-expressed with *PLCG2* (413 genes) identified significant GO terms, including response to unfolded protein and lipopolysaccharide, regulation of small GTPase-mediated signal transduction, regulation of I-kappaB kinase/NF-kappaB signaling, and regulation of cell population proliferation, as shown in Fig. 5c.

## Discussion

**Fig. 6:**
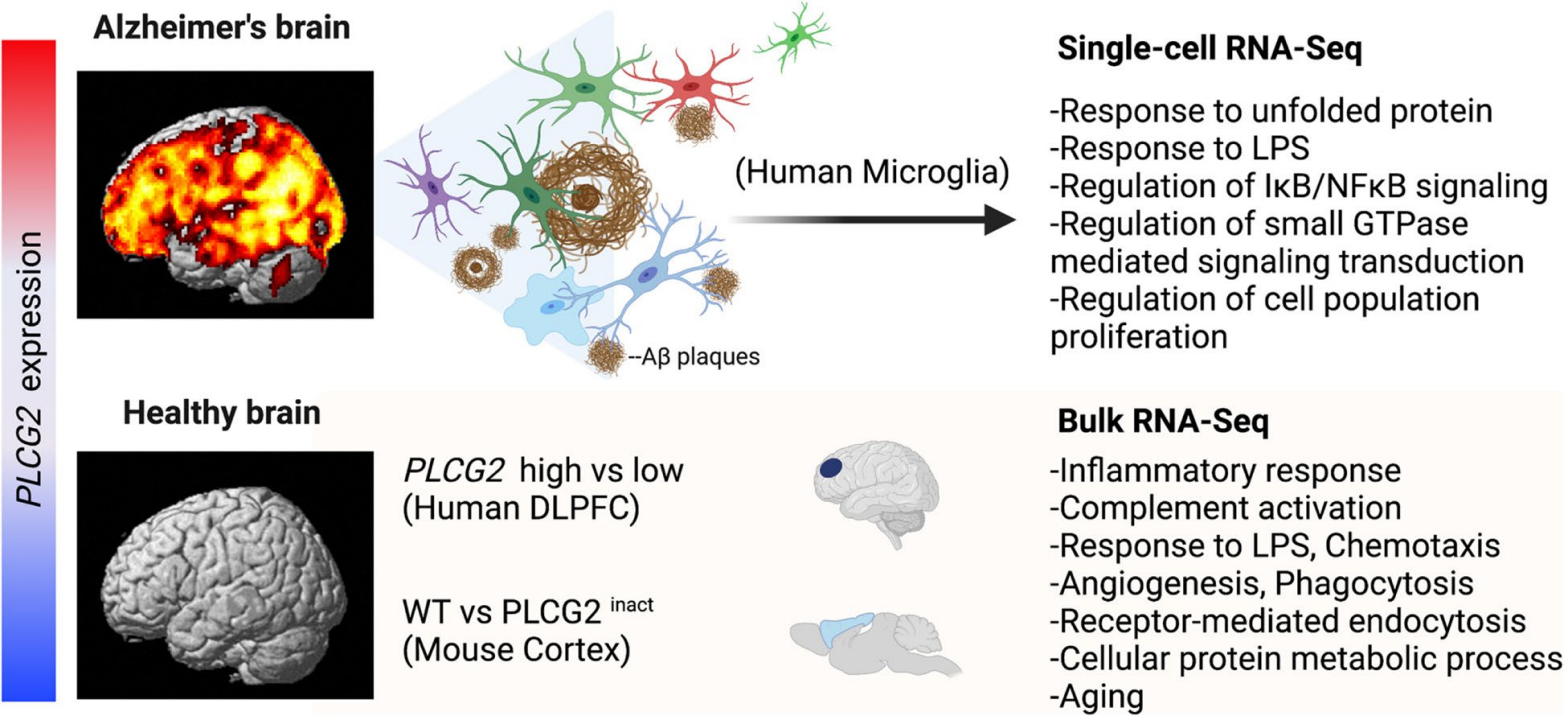
Overview of our findings for *PLCG2* in the brain and plaque-associated microglia*. PLCG2* expression was increased in LOAD patients and was associated with plaque density. *PLCG2* was increased exclusively in microglia in the AD mouse model. The inflammation-related pathways were identified in human and *Plcg2*-inactivated mouse brains and *PLCG2* co-expression network analysis of human microglia single-cell RNA-Seq

Although *PLCG2* variants were previously identified to be associated with AD risk [9, 32, 33], the role of *PLCG2* in AD is still poorly understood. Here, we report that *PLCG2* expression is increased in several brain regions of LOAD patients and that increased *PLCG2* expression levels positively correlate with both brain amyloid plaques and expression levels of microglial marker genes (*AIF1* and *TMEM119*) (Fig. 6) [34–36]. These results highlight an important relationship between amyloid plaques and *PLCG2* expression, further supported by increased expression levels of *Plcg2* throughout the disease progression in 5xFAD mice, a well-studied model of amyloid pathology [37]. Moreover, expression levels of *PLCG2* were significantly associated with expression levels of microglia-specific marker genes (*AIF1* and *TMEM119*), which we validated in 5xFAD mice, showing that increased *Plcg2* expression was induced by amyloid plaques and the increase reverted to control levels after significantly reducing microglia with the colony-stimulating factor-1 antagonist PLX5622. Interestingly, we did not observe an increase in *Plcg2* expression in a 12-month tauopathy mouse model (P301S), which exhibits robust microgliosis, suggesting that the increase of *Plcg2* might result from an association between microglia and amyloid plaques, rather than microgliosis.

Accumulating evidence suggests that the modulation of the TREM2/PLCG2 signaling pathway is a potential therapeutic candidate for AD [16]; however, the role of *PLCG2* and the signaling networks associated with *PLCG2* in the brain remain unclear. We identified differentially expressed genes and pathways by performing differential gene expression analysis on bulk RNA-Seq data from CN with 25th-percentile *PLCG2* gene expression levels (low) and 75th-percentile *PLCG2* gene expression levels (high). These genes and pathways are related to the inflammatory response, cytokine-mediated signaling, regulation of complement activation, chemotaxis, response to lipopolysaccharide, regulation of I-kappaB/NF-kappaB signaling, and response to interleukin-1. A previous study of human induced pluripotent stem cell-derived microglia-like (iMG) cells revealed that *PLCG2* is required to induce proinflammatory signaling, a finding recapitulated by the results of our pathway analysis [38], reinforcing the role of *PLCG2* in the inflammatory response. Our findings in humans were validated in mice with inactivated *Plcg2* expression (PLCG2 ^inact^)*,* which reduced expression levels of AD-associated microglial genes [39, 40], including *CLEC7A*, *C3*, and *PIK3R5,* in comparison with expression levels of these genes in WT mice. Importantly, the reduction of the expression of AD-associated microglial genes was not accompanied by changes in microglial cell coverage or morphology as determined by immunostaining, indicating that the observed gene expression changes were not due to the increased numbers of microglia cells. These results suggest a potential role for *PLCG2* in inflammation-related pathways.

A previous single-cell RNA-sequencing study of human CN, MCI, and AD microglia identified that about 80% of all microglia fell into a homeostatic subtype [24]. We performed a co-expression network analysis using microglia single-cell RNA-Seq data with fresh isolated cells from MCI and AD donors to identify working partners of *PLCG2* under disease conditions that may have potential roles as microglia-directed therapeutic targets. This analysis identified *IL1B* as one of the top 20 genes included in the *PLCG2* co-expression network, recapitulating the result of a previous study in which *IL1B* expression was downregulated in *PLCG2* knock-out iMG cells after zymosan challenge [38]. Furthermore, we performed a gene-set enrichment analysis of the top 20% of genes included in the *PLCG2* co-expression network. The identified pathways were related to the response to unfolded protein, regulation of small GTPase mediated signal transduction, regulation of I-kappaB kinase/NF-kappaB signaling, response to lipopolysaccharide, and regulation of cell population proliferation. These findings suggest a potential role for *PLCG2* in AD microglia, which may allow us to develop a novel therapeutic strategy targeting *PLCG2* or the signaling networks associated with it.

## Conclusions

In conclusion, *PLCG2* expression is increased in several brain regions in LOAD patients and significantly correlates with brain amyloid burden in LOAD patients and AD model mice. Specifically, 5xFAD amyloid pathology mice, we observed a disease progression-dependent increase in *Plcg2* expression. The results of this study show that 5xFAD and *Plcg2*-inactivation mouse models are appropriate for studying *PLCG2* in AD. We believe that future studies of genetic mouse models are needed to further clarify the role of *PLCG2* in plaque-associated microglia and to determine whether decreased *PLCG2* expression in plaque-associated microglia favors disease exacerbation or attenuation. Moreover, investigating how *PLCG2* modulates microglia phenotypes in AD may identify novel therapeutic strategies for microglia-targeted AD therapies.

## Supplementary Information

**Additional file 1: Table S1**: PLCG2 expression levels in different brain regions. **Table S2**: Levels of plaque mean density, and the expression levels of *PLCG2, AIF1*, and *TMEM119* in MSBB cohort. **Table S3**: Differential expression analysis from CN with 25^th^-percentile PLCG2 gene expression levels and 75^th^-percentile PLCG2 gene expression levels. **Table S4**: Differential expression analysis of mouse data. **Table S5**: Co-expression analysis of PLCG2 in human microglia scRNA-seq analysis.

**Additional file 2: Figure S1**: Plcg2 expression does not change in a 12-month tauopathy mouse model, despite microgliosis. **Figure S2**: Differential expression analysis in Plcg2 inactivation mice identified inflammation-related pathways. 

## Data Availability

RNA-Seq data were downloaded from the Accelerating Medicines Partnership for Alzheimer’s Disease (AMP-AD) Consortium (https://adknowledgeportal.synapse.org/Explore/Programs/DetailsPage?Program=AMP-AD): The raw data from ROS/MAP cohort (https://www.synapse.org/#!Synapse:syn8456638) [17], Mayo cohort (https://www.synapse.org/#!Synapse:syn8466816) [18], and MSBB cohort (https://www.synapse.org/#!Synapse:syn8485017) [19] are accessible through the Synapse database (https://www.synapse.org/). The raw data from Olah et al. are accessible through Synapse (https://www.synapse.org/#!Synapse:syn21438358) [24]. The NanoString data reported in this paper are available in the Gene Expression Omnibus (GEO) database with accession ID GSE195650, https://www.ncbi.nlm.nih.gov/geo/query/acc.cgi?acc=GSE195650 [41]. Our single-cell-based transcriptomic data analysis from human microglia are available in the form of a browsable platform at https://andyptsai.shinyapps.io/ROSMAP_micorglia_scRNAseq_explorer/.
