# Supplementary material for: PLCG2 is associated with the inflammatory response and is induced by amyloid plaques in Alzheimer’s disease
Source: Genome Med. 2022 Feb 18;14:17. doi: 10.1186/s13073-022-01022-0 (PMC8857783; doi:10.1186/s13073-022-01022-0)
Supplement: Supplementary file 2 — Additional file 2: Figure S1: Plcg2 expression does not change in a 12-month tauopathy mouse model, despite microgliosis. Figure S2: Differential expression analysis in Plcg2 inactivation mice identified inflammation-related pathways. [file 13073_2022_1022_MOESM2_ESM.pptx]

## Slide 1
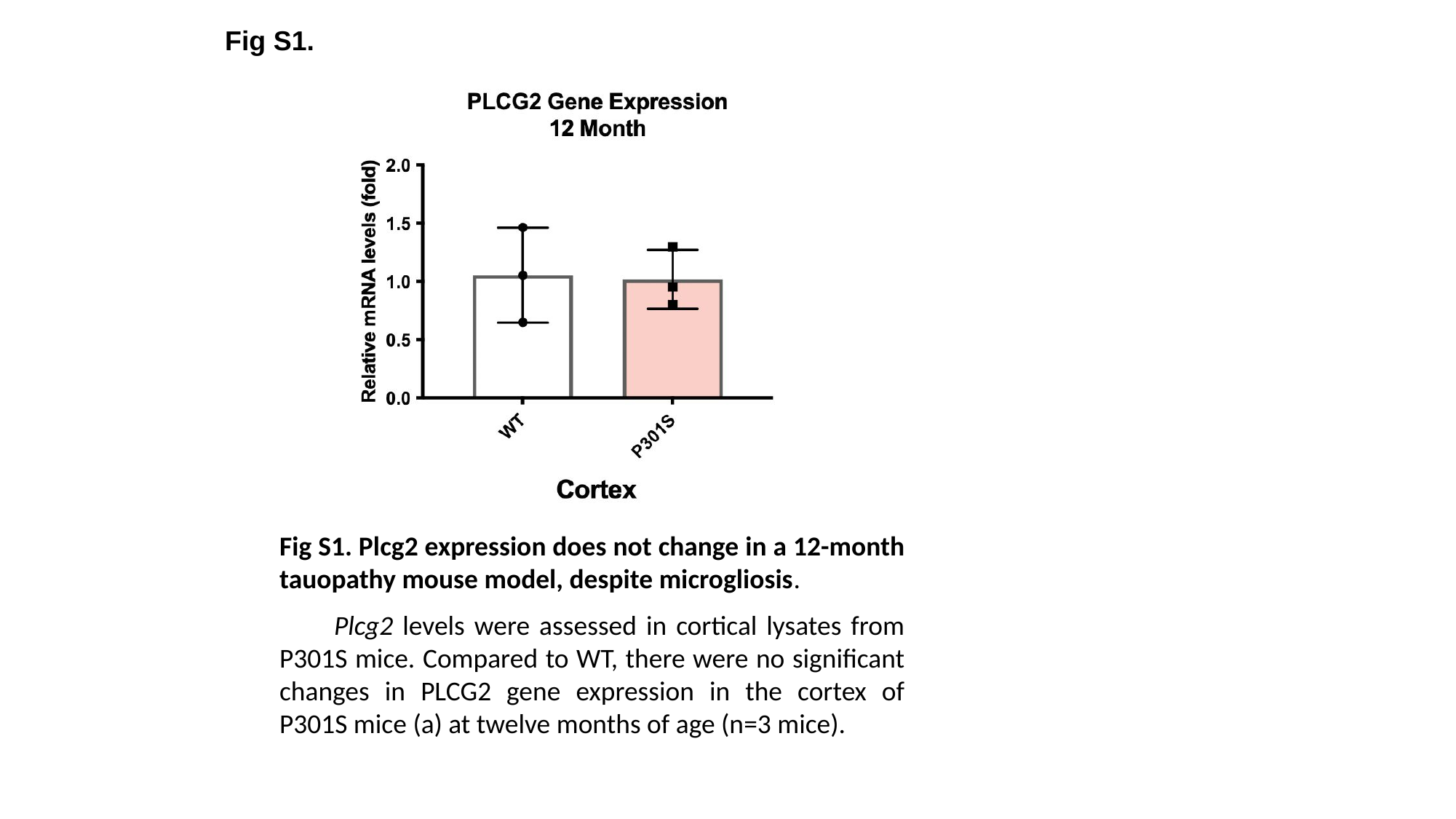

Fig S1.
Fig S1. Plcg2 expression does not change in a 12-month tauopathy mouse model, despite microgliosis.
Plcg2 levels were assessed in cortical lysates from P301S mice. Compared to WT, there were no significant changes in PLCG2 gene expression in the cortex of P301S mice (a) at twelve months of age (n=3 mice).

## Slide 2
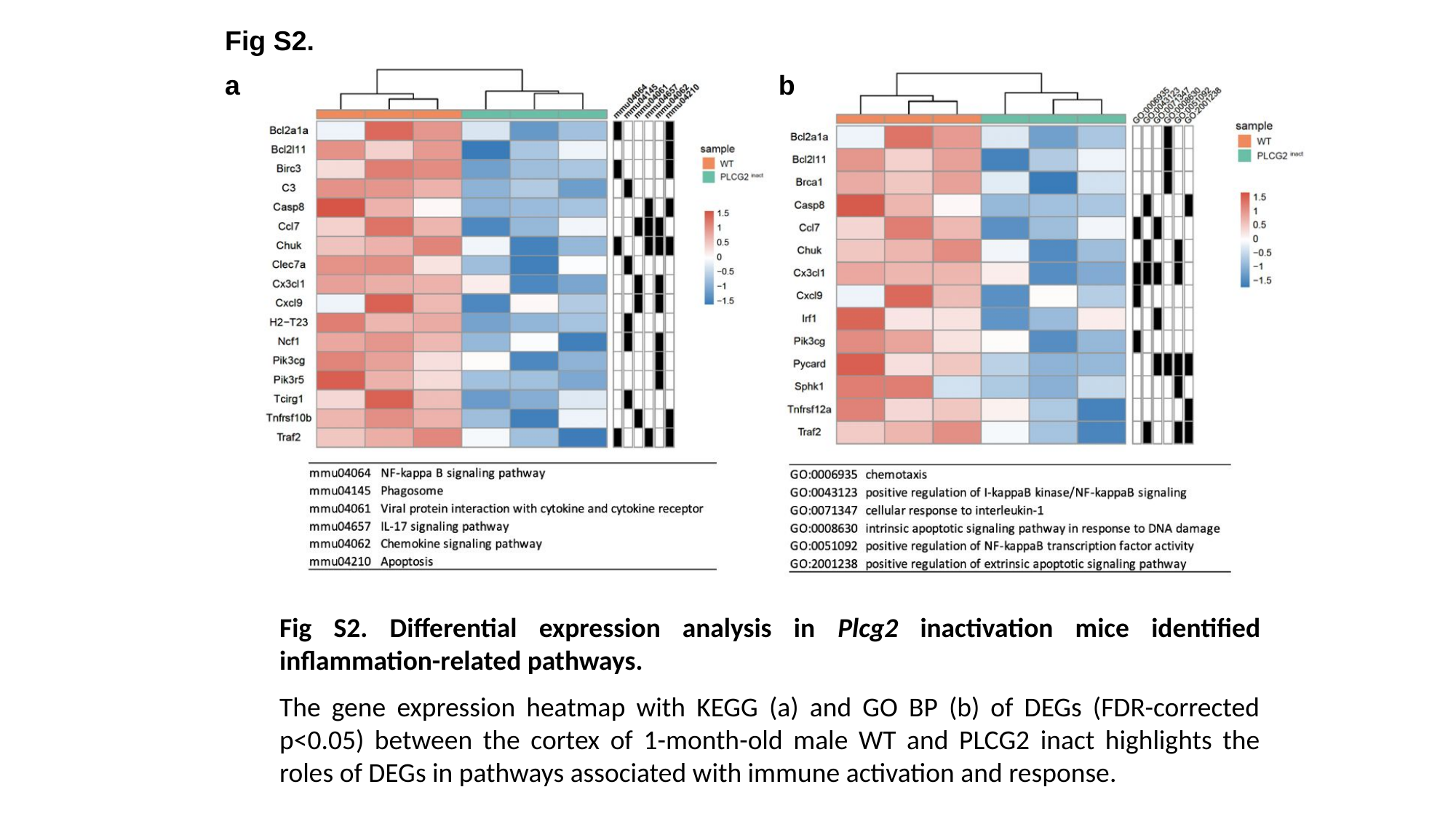

Fig S2.
a
b
Fig S2. Differential expression analysis in Plcg2 inactivation mice identified inflammation-related pathways.
The gene expression heatmap with KEGG (a) and GO BP (b) of DEGs (FDR-corrected p<0.05) between the cortex of 1-month-old male WT and PLCG2 inact highlights the roles of DEGs in pathways associated with immune activation and response.
